# Absence of Anomalous Electron–Phonon Coupling in the Near-Ambient Gap Temperature Renormalization of CsPbBr_3_ Nanocrystals

**DOI:** 10.1021/acs.jpcc.4c06265

**Published:** 2024-12-19

**Authors:** Shima Fasahat, Benedikt Schäfer, Kai Xu, Nadesh Fiuza-Maneiro, Sergio Gómez-Graña, M. Isabel Alonso, Lakshminarayana Polavarapu, Alejandro R. Goñi

**Affiliations:** † Institut de Ciència de Materials de Barcelona, ICMAB-CSIC, Campus UAB, 08193 Bellaterra, Spain; ‡ CINBIO, Materials Chemistry and Physics Group, Department of Physical Chemistry, Universidade de Vigo, Campus Universitario Lagoas Marcosende, 36310 Vigo, Spain; § ICREA, Passeig Lluís Companys 23, 08010 Barcelona, Spain

## Abstract

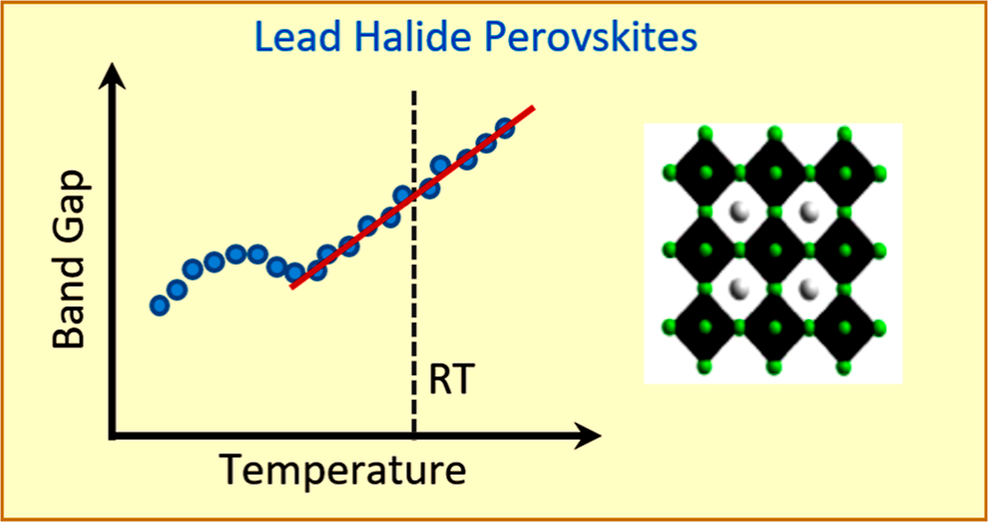

Metal halide perovskites exhibit a fairly linear increase of the bandgap with increasing temperature, when crystallized in a tetragonal or cubic phase. In general, both thermal expansion and electron–phonon interaction effects contribute equally to this variation of the gap with temperature. Herein, we have disentangled both contributions in the case of colloidal CsPbBr_3_ nanocrystals (NCs) by means of photoluminescence (PL) measurements as a function of temperature (from 80 K to ambient) and hydrostatic pressure (from atmospheric to ca. 1 GPa). At around room temperature, CsPbBr_3_ NCs also show a linear increase of the bandgap with temperature with a slope similar to that of the archetypal methylammonium lead iodide (MAPbI_3_) perovskite. This is somehow unexpected in view of the recent observations in mixed-cation Cs_*x*_MA_1–*x*_PbI_3_ single crystals with low Cs content, for which Cs incorporation caused a reduction by a factor of 2 in the temperature slope of the gap. This effect was ascribed to an anomalous electron–phonon interaction induced by the coupling with vibrational modes admixed with the Cs translational dynamics inside the cage voids. Thus, no trace of anomalous coupling is found in CsPbBr_3_ NCs. However, we managed to show that the linear temperature renormalization exhibited by the gap of CsPbBr_3_ NCs is shared with most metal halide perovskites, due to a common bonding/antibonding and atomic orbital character of the electronic band-edge states. In this way, we provide a deeper understanding of the gap temperature dependence in the general case when the A-site cation dynamics is not involved in the electron–phonon interaction.

## Introduction

Colloidal lead halide perovskite nanocrystals (NCs) have attracted significant attention in recent years as promising candidates for next-generation optoelectronics.^1−3^ This is due to their high photoluminescence (PL) quantum yields and the tunability of energy gap concerning different parameters including dimensionality, composition, as well as external stimuli like temperature and pressure.^4−6^ Metal halide perovskites (MHPs) are materials with ABX_3_ general formula, where A corresponds to an organic or inorganic cation like methylammonium (MA), formamidinium (FA) or Cs, B is a metallic bivalent cation such as Pb or Sn and X is a halide anion like I, Br, Cl. Mixed compositions in either A, B, or X positions provide optical properties tunability. As an example, mixed-anion compounds can be obtained via ionic exchange leading to the substitution of different halides.^7^ This process is particularly effective in NCs.^8^ Common structural behavior of these perovskites is that at high temperatures the crystal phase is cubic (C) and with decreasing temperature the phase transforms to tetragonal (T) and then orthorhombic (O).^9^ In the particular case of bulk CsPbBr_3_, the phase transition from C to T takes place at around 400 K, and at around 360 K the transition from T to O phase occurs.^10^ In the C and T phases, A-site cations are enclosed in BX_6_ octahedral cages where they can freely move; depending on the chemical species this movement includes translation, rotation, and libration inside the cage voids. Recent studies highlighted the importance of the A-site cation dynamics at different temperatures and pressures in the structural behavior of MHPs.^6,9^ In particular, the fast roto-translational dynamics in cubic and tetragonal phases is fully or partially unfolded. In contrast, A-site cations are locked in specific positions and orientations inside the voids in less symmetric orthorhombic phases.^11^ Regarding CsPbBr_3_ NCs, the A-site cation dynamics also affects the structural behavior as compared to bulk. High-resolution transmission electron microscopy (TEM) studies of CsPbBr_3_ single nanocubes at room temperature, in combination with results of simulations, hint at a supercooling of the cubic phase for NC sizes smaller than 10 nm.^12^ That means that NCs are still cubic at temperatures much lower than the transition temperature for bulk (360 K), including room temperature. In contrast, in the case of larger NCs, they exhibit a minority of the O phase that coexists with the C.^12^

A precise understanding of the band gap energy in semiconductors and its dependence on external parameters including temperature and pressure is essential for various optoelectronic applications. Regarding the behavior of the fundamental gap with temperature, most perovskite NCs in the tetragonal and cubic phase exhibit a linear dependence with a positive slope and a typical value of ca. 0.2 meV/K. Examples are MAPbI_3_,^13−15^ MAPbBr_3_,^16−18^ FAPbI_3_,^14,19,20^ FAPbBr_3_,^13,16,21,22^ CsPbI_3_,^13,23−26^ and CsPbBr_3_.^13,16,23,25,27−29^ A notorious exception are CsPbCl_3_ NCs and thin films,^13,25,30^ which display an outspoken negative slope around room temperature. In this respect, a quantitative analysis of the relative weight of thermal expansion and electron–phonon coupling on this temperature slope is still lacking, in particular for CsPbBr_3_ NCs.

The energy gap dependence on temperature is bipartite. One point of consideration is the change of electronic band structure due to the variation of the lattice potential, which is the outcome of contraction/expansion of the lattice induced by temperature, known as the thermal expansion term (TE). The other contribution is the effect of lattice vibrations on the lattice potential that leads to the energy renormalization of the electronic band structure, generally stronger at higher temperatures, called electron–phonon coupling term (EP). The variation of the gap concerning temperature reads as^31^
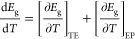
1

In the case of MAPbI_3,_ it was demonstrated that TE and EP terms possess almost equal weight in the temperature dependence of the gap.^31^ In what follows, we will show, according to a literature survey that the linear temperature dependence of the gap in cubic and tetragonal phases is a general behavior for most of the perovskites. Nevertheless, there is a case in which a small amount of Cs incorporated in MAPbI_3_ leads to a reduction of the slope by a factor of 2.^32^ This reduction was interpreted as due to an anomalous electron–phonon coupling, involving the interaction of electrons with the movement of the Cs inside the cage voids in synchrony with dynamic octahedral tilting. A motivation to investigate pure CsPbBr_3_ NCs was to find out whether an anomalous behavior can be linked in general to Cs dynamics and to rationalize the different contributions to the temperature dependence of the gap in MPHs.

In this work, we studied the dependence of the energy gap of CsPbBr_3_ NCs on temperature and pressure by means of PL experiments. We observe a linear temperature dependence of the energy gap of these NCs in the range around room temperature, for which the A-site cation dynamics (Cs in this case) is fully unfolded.^9^ High-pressure experiments allowed us to disentangle TE and EP terms, leading to a full understanding of the temperature dependence of the bandgap. For the NCs, we observed a similar dependence as in bulk and other perovskites, with the TE and EP terms having similar magnitude as for MAPbI_3_. Although the studied samples are pure Cs perovskites, there was no indication of an additional electron–phonon coupling mechanism of any kind that could be attributed to the motion of Cs together with dynamic octahedral tilting. In other words, we did not detect any anomalous electron–phonon coupling in the case of CsPbBr_3_ NCs. On the contrary, CsPbBr_3_ NCs show the common behavior of most MHPs, i.e. a linear gap temperature renormalization with a positive slope ranging from ca. 0.15 to 0.4 meV/K. This behavior, which we show results from the bonding/antibonding character and the atomic orbital nature of the band-edge states, is quite general and considered normal in MHPs according to a survey carried out and reported here for the gap pressure and temperature coefficients of 19 lead halide perovskites.

## Methods

### CsPbBr_3_ NCs Synthesis

In a typical synthesis, 15 mL of octadecene, 1.5 mL of oleic acid, and 1.5 mL of oleylamine and the precursor powders (1 mmol of Cs_2_CO_3_ and 3 mmol of PbBr_2_) were loaded in a 50 mL glass vial. Then, the reaction medium was subjected to tip-ultrasonication (SONOPULS HD 3100, BANDELIN) for 30 min at a power of 30 W. During this time, a color change of the reaction mixture can be observed to yellow indicating the formation of CsPbBr_3_ perovskite NCs. The solution was purified by centrifugation (10,000 rpm, 10 min) and the pellet was redispersed in 6 mL of hexane. Finally, the solution was centrifuged again (5000 rpm, 10 min) to remove the large particles. The supernatant was collected to obtain CsPbBr_3_ NCs. The produced NCs were analyzed by TEM using a FEI Tecnai G2 F20 HR(S) microscope.

### Temperature and Pressure-Dependent PL Experiments

For excitation of the PL spectra a violet laser (405 nm) was used, employing a very low laser power of ca. 2 μW (power density < 15 W/cm^2^) to prevent any photodegradation of the samples.^33,34^ Spectra were recorded using a 20× long working distance objective with NA = 0.35 (laser spot of ca. 4 μm in diameter) and dispersed with a high-resolution LabRam HR800 grating spectrometer equipped with a charge-coupled device detector. PL spectra were corrected for the spectral response of the spectrometer by normalizing each spectrum using the detector and 600 grooves/mm grating characteristics. Temperature-dependent PL measurements on CsPbBr_3_ NCs were carried out by decreasing the temperature between 300 and 80 K in steps of 5 K using liquid nitrogen in a gas flow cryostat from CryoVac with optical access that fits under the microscope of the Raman setup. The high-pressure PL measurements in the range up to ca. 1 GPa were performed at room temperature by employing a gasketed diamond anvil cell (DAC). Anhydrous propanol was used as a pressure-transmitting medium which ensures perfect hydrostatic conditions up to 4.2 GPa.^35^ For loading the DAC a droplet of CsPbBr_3_ NC solution was drop-casted on one of the diamonds of the DAC and allowed to dry. Then, a few ruby balls were added for pressure calibration.^36^ After the preindentation, the thickness of the gasket was 120 μm. Afterward, a hole of ca. 250 μm was drilled with a sparkgap machine from EasyLab. This allowed us to adjust the pressure with the DAC in steps of less than 0.05 GPa, mainly at very low pressures (below 0.2 GPa). For this purpose, an electric motor drive was used to change the pressure in a continuous manner and at low speed (by ca. 0.05 GPa/min).

## Results and Discussion

### Temperature-Dependent PL Measurements

**Figure 1:**
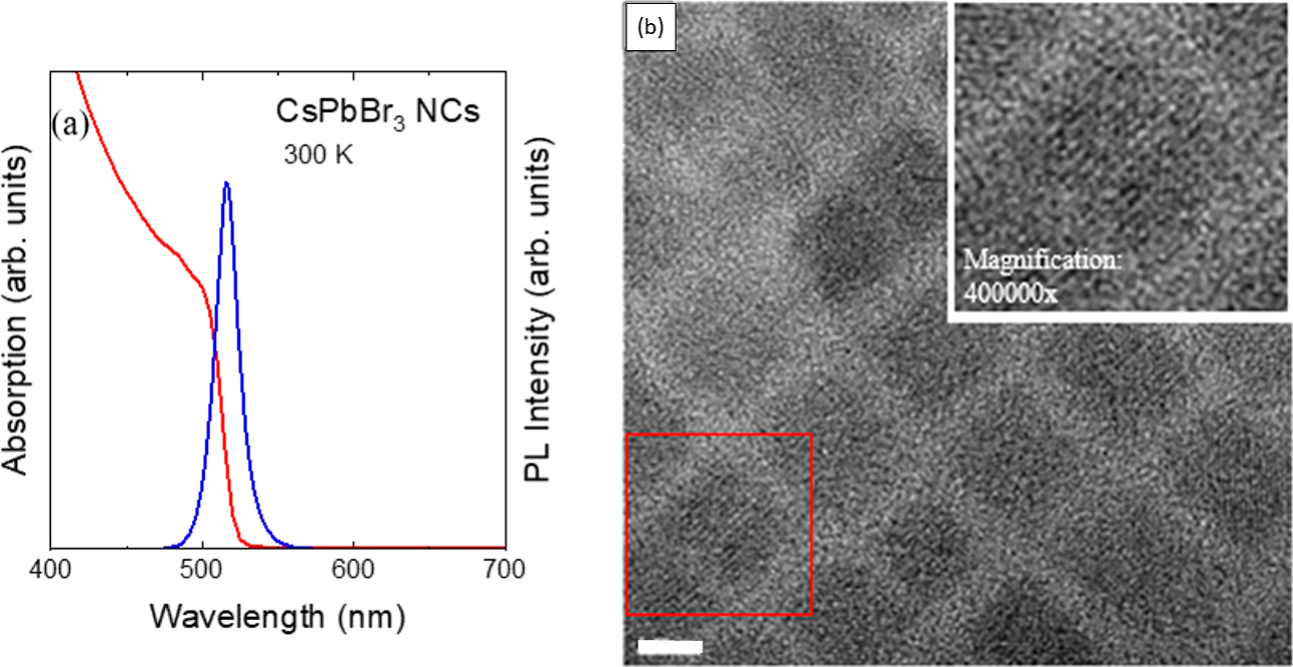
(a) Absorption and PL spectra of CsPbBr_3_ NCs at room temperature. (b) High-resolution TEM micrograph of CsPbBr_3_ NCs. The scale bar corresponds to 5 nm.

The as grown NCs exhibit at room temperature a fairly intense PL and a sharp absorption edge, as depicted in Figure 1a. The structural characterization of CsPbBr_3_ NCs via high-resolution TEM indicates that the sample is fairly crystalline. Figure 1b shows a TEM image of CsPbBr_3_ NCs with ordered cubic shapes. The high resolution TEM images allowed us to measure the side lengths of the nanocubes precisely. The NC size was calculated by taking the average lateral length for each of the cuboids. The corresponding histogram of NC sizes is shown in Figure S1 of the Supporting Information, indicating a relatively narrow size distribution with an average edge length of 8 nm.

As mentioned before, bulk CsPbBr_3_ crystallizes at room temperature in an orthorhombic phase.^10^ However, recent work on CsPbBr_3_ nanocrystals (NCs) shows that the structural behavior of NCs is size-dependent. In fact, crystals exhibit a more cubic phase rather than orthorhombic when their size is smaller than 10 nm,^12^ similar to the NCs used in this study. This is due to the fact that strain relaxation encourages the stabilization of the cubic α phase at room temperature when the Cs dynamics is unfolded.^9,10^ The Raman spectrum of CsPbBr_3_ NCs (Figure S2 of the Supporting Information) supports this statement. For completeness we point out that NCs produced by other synthetic methods such as ball milling^37^ or hot injection^38^ do crystallize in the orthorhombic phase. Nevertheless, the stabilization of the cubic phase down to temperatures much lower than the thermodynamic transition temperature has been reported in mixed-cation lead and tin iodide thin films.^39^ Responsible for such a supercooling of the cubic phase is the impact of thermally induced dynamic lattice distortions mediated by dynamic steric interaction, occurring at temperatures for which the A-site cation dynamics is fully unfolded.^9^ Furthermore, recent results of ab initio quantum dynamics simulations clearly indicate that structural disorder is as important as dynamic disorder (related to the Cs dynamics) for the stabilization of the cubic phase.^40^ As perovskite NCs consist of a fairly ordered, single crystalline core and a structurally disordered shell, with decreasing temperature the shell might provide anchoring points for the cubic phase, stable at the temperature at which the NCs are produced. This would further help to supercool the cubic phase delaying the transition into the orthorhombic phase. Because the surface-to-volume ratio increases with decreasing NC size, this would also explain why smaller NCs are mostly cubic at room temperature, as demonstrated by high-resolution TEM measurements.^12^ For these reasons we believe that the cubic phase is the stable one in the temperature range between 250 and 300 K (see discussion on the linearity range below).

**Figure 2:**
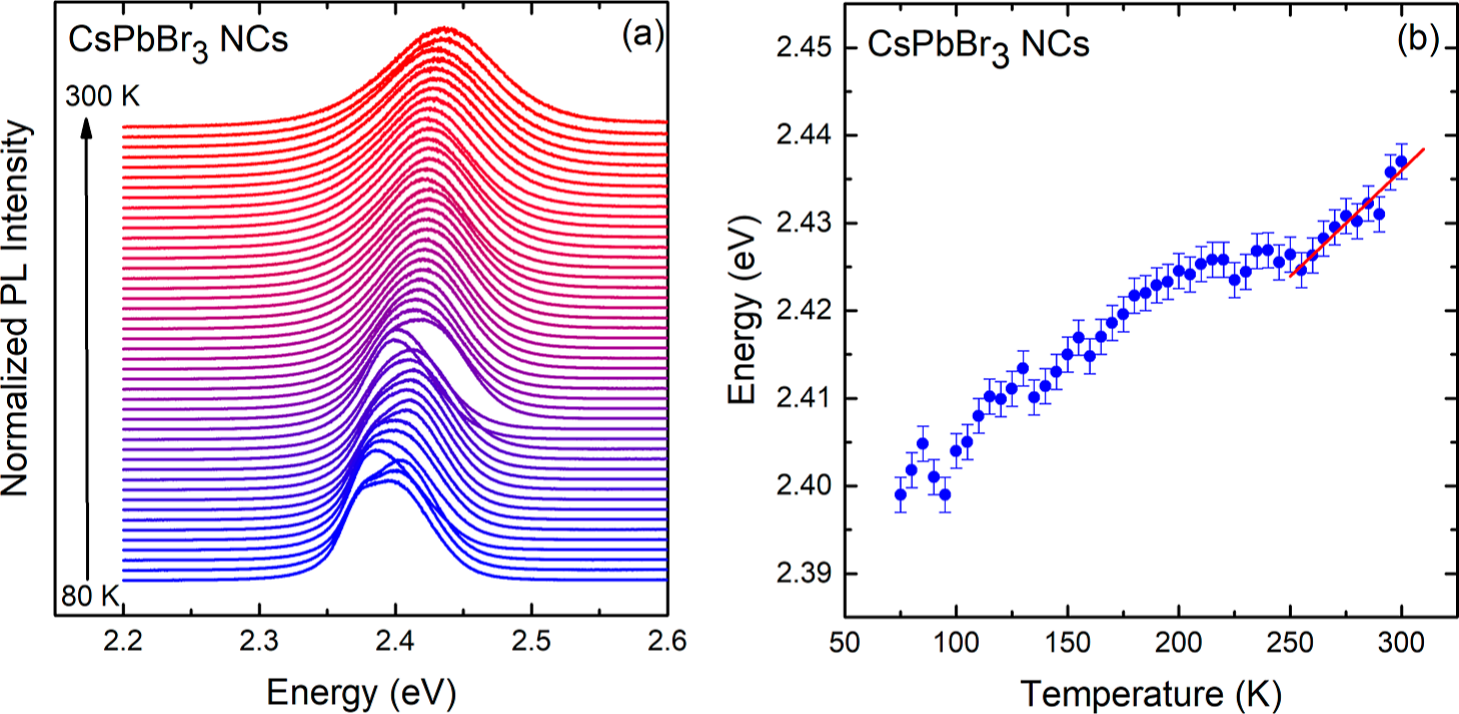
(a) PL spectra of CsPbBr_3_ NCs recorded at various temperatures ranging from 80 to 300 K, in 5 K increments. The spectra were normalized to their peak intensity and vertically offset for better visualization. (b) Temperature dependence of energy gap of CsPbBr_3_ NCs (blue symbols). The red line represents a linear fit to the data points around room temperature.

Figure 2a displays the PL spectra of CsPbBr_3_ NCs recorded at different temperatures from 80 to 300 K. These spectra were normalized to their absolute maximum intensity and vertically shifted to ease the comparison. Evolution of spectra in this temperature range exhibits an almost uniform behavior without noticeable phase transition.^41^ The occasional small shift of the temperature-dependent spectra (within the width of the PL peak) is just due to slight inhomogeneities of the sample and the fact that the excitation of the PL does not always occur on the same spot. The main PL peak which is the result of the radiative recombination of free excitons,^42^ shows a small progressive shift to higher energies by increasing the temperature.

In order to analyze the PL spectra, a Gaussian–Lorentzian cross-product function was employed to describe the main PL peak. It is the same method utilized previously for analyzing PL peaks of MAPbI_3_,^31,43^ Cs_*x*_MA_1–*x*_PbI_3_ (*x* = 0.05, 0.1) perovskites,^32^ and a series of FA_*x*_MA_1–*x*_PbI_3_ solid solutions.^42,44^ There are four adjustable parameters in this cross-product function which are: the amplitude prefactor *A*, the peak maximum position *E*_0_, the full width at half-maximum (fwhm) Γ, and a line shape weight *s* (0 for pure Gaussian, 1 for pure Lorentzian).^45^ PL peaks in these spectra have *s* values in the range of 0.4–0.6. Peak widths at temperatures between 80 and 180 K are dominated by inhomogeneous (Gaussian) broadening, whereas from 185 to 300 K the behavior of the line width reveals that homogeneous (Lorentzian) broadening takes gradually over. This is due to the size distribution and the small average size of the NCs. The spectra plotted in Figure 2a show the red shift and sharpening of the peaks with decreasing temperature. The position of the maximum of the PL peaks *E*_0_ can be considered to represent the energy of the bandgap, because of the small exciton binding energy of around 15 meV.^44^ The temperature dependence of the energy gap is thus plotted in Figure 2b. As mentioned previously, the behavior of *E*_0_ with increasing temperature is quite steady with a total increase of almost 45 meV from 80 to 300 K.

In this work, however, we will focus on the temperature range from approximately 250 to 300 K, in which *E*_0_ exhibits a markedly linear temperature dependence with a positive slope, as indicated by the red line in Figure 2b. The slope turns out to be 2.3(5) × 10^–4^ eV/K, a value similar to that previously obtained for MAPbBr_3_,^18^ Cs_*x*_MA_1–*x*_PbI_3_^32^ and FA_*x*_MA_1–*x*_PbI_3_^42^ in a similar temperature range around room temperature. The observation of such linearity in the temperature dependence of the gap is of fundamental and practical importance. On the one hand, the linear gap dependence is a consequence of the NCs being cubic and, viceversa, in the cubic phase, octahedral tilting plays no role and the temperature behavior of the perovskite gap is solely determined by the effects of bond stretching. The latter strictly depend on the bonding/antibond and atomic orbital character of the electronic states at the top of the valence band and the bottom of the conduction band, typically leading to a linear opening of the gap with increasing temperature.^43,46^ Octahedral tilting, however, causes always an increase of the electronic gap, as a result of the effects of bond bending. The hybridization of the Pb 6p orbitals with the s orbitals of the corresponding halide anions imposes a serious constraint over the halide-Pb-halide bond to be straight. Any temperature (or pressure) induced tilting of the corner-sharing PbX_6_ octahedrons gradually reduces the X-Pb-X bond angle below 180° with the concomitant blue shift of the gap energy.^47−51^ Usually, the bond-bending effects (octahedral tilting) compete with those due to bond stretching, thus leading to an outspoken nonlinear temperature or pressure dependence of the gap. Please notice that the bowing in the gap temperature dependence of the CsPbBr_3_ NCs, setting in below ca. 250 K (see Figure 2b), might be an indication of the structural phase transition from cubic to the thermodynamically favored orthorhombic phase.^37,38^

At this point we would like to emphasize that in the whole linearity range, the first derivative of the gap over temperature is a constant, hence, both the TE and EP terms must be temperature independent as well. This is the principal reason for restricting the analysis of the gap temperature renormalization solely to the limited temperature range of the near-ambient linearity. Although the high pressure experiments with the DAC can currently be performed only at room temperature, the value of the TE term determined that way is still valid for the entire temperature range of the near-ambient linearity, due to the constancy of the temperature and pressure derivatives of the gap. Outside the linearity regime, however, a proper analysis of the temperature dependence of the gap would require to carry out high pressure experiments at different temperatures, since the gap pressure coefficient and, thus, the TE term are strongly dependent on temperature (see, for instance, the results obtained for the low-temperature orthorhombic phase of MAPbI_3_^52^). Consequently, special equipment would be necessary, like a bath cryostat allocating the diamond anvil cell and the use of helium as pressure transmitting medium, that needs cryogenic loading as well. All this is, unfortunately, out of the scope of this work.

### Disentangling Thermal Expansion and Electron–Phonon Coupling Terms

As described in eq 1, two terms build the derivative of the energy gap with temperature: thermal expansion and electron–phonon coupling. The former can be identified with the effect of external hydrostatic pressure on the gap, which is directly related to the shrinkage of the lattice with decreasing temperature. Thus, the following equation gives the thermal expansion term
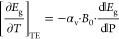
2where α_v_ is the volumetric thermal expansion coefficient, *B*_0_ refers to the bulk modulus and the last magnitude  corresponds to the pressure coefficient of the energy gap, which will be assessed through high-pressure experiments.^53^ Most direct band gaps in conventional semiconductors have a positive pressure coefficient at room temperature,^46^ therefore, according to eq 2, TE reduces the energy gap with increasing temperature. Lead halide perovskites, however, show the opposite behavior partly because the sign of the TE term is reversed in view of the fact that the sign of the gap pressure coefficient  is negative.^54^

**Figure 3:**
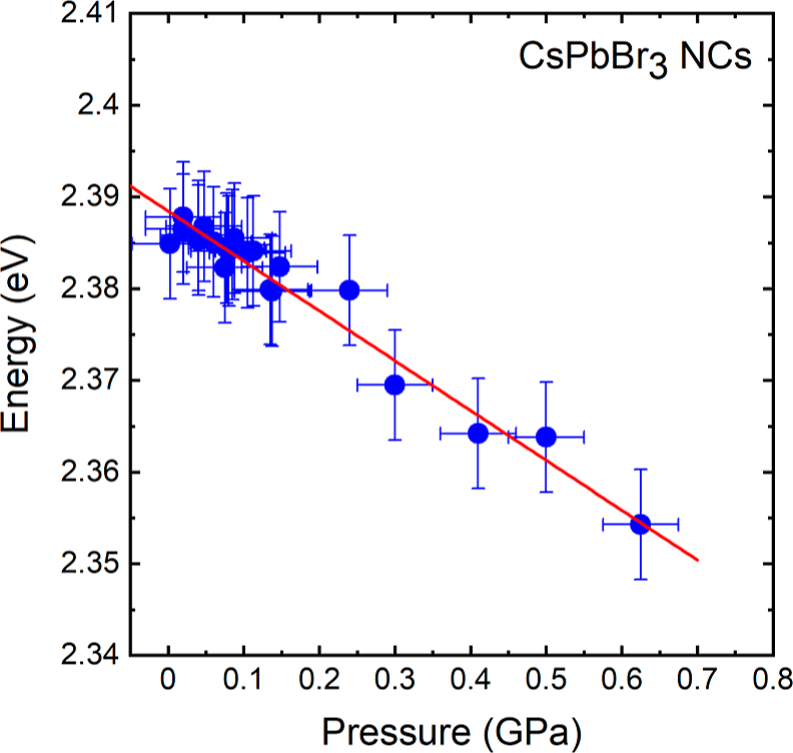
Pressure dependence of energy gap of CsPbBr_3_ NCs recorded at room temperature.

PL spectra of CsPbBr_3_ NCs were acquired at different pressures up to ca. 1 GPa (see Figure S3 of the Supporting Information). As discussed previously, the center energy of the PL peaks is representative of the energy gap. Figure 3 shows the pressure dependence of the gap at room temperature. The straight red line through the data points is the result of a linear fit and the slope gives directly the experimental value of the pressure coefficient, namely −0.055(15) eV/GPa. Here we consider only the data corresponding to the stability range of the cubic phase of the NCs (*P* < 0.7 GPa), because the gap pressure derivative that enters eq 2 corresponds precisely to the initial slope of the gap-vs-pressure curve for an infinitesimal variation of the pressure close to ambient conditions (see Section S3 of the Supporting Information for details). To calculate the TE term according to eq 2, we used the values of volumetric expansion coefficient and bulk modulus reported in the literature such as 1.14(10) × 10^–4^ K^–1^,^55^ and 21(3) GPa,^56^ respectively. The obtained TE term amounts to 1.30(40) × 10^–4^ eV/K. This is almost half of the whole measured temperature slope of the gap. This means that the other half is related to the contribution from the interactions between electrons and phonons, the EP term, as explained below.

The renormalization of the gap energy due to electron–phonon interaction is mainly infuenced by phonon modes corresponding to peaks in the phonon density of states (DOS).^57^ Indeed, this forms the basis of the Einstein oscillator model proposed by Cardona and co-workers,^58−60^ to describe the EP term in a practical way by considering effective electron–phonon interaction coefficients *A*_*i*_ for phonons with an average frequency ω_*i*_, derived from peaks in the phonon DOS. The electron–phonon correction to the gap thus reads as
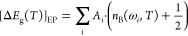
3Here, *n*_B_ denotes the Bose–Einstein phonon occupation factor and shows the explicit temperature dependence of the renormalization. The coefficient *A*_*i*_ is temperature-independent but it may be different depending on the particular average frequency and the mass of the atomic species involved in the vibration. This specific aspect can be taken into account in materials with two atoms per unit cell that have significantly different masses, such as the cuprous halides, by using a two-oscillator model.^59,60^ In that case, this is justified by the phonon DOS showing two peaks: one at the average frequency of the acoustic phonon branches at the Brillouin zone edges (corresponding to vibrations of the heavier atom), and another representing the optical phonon contribution (corresponding to vibrations of the lighter atom). However, this scheme cannot be directly applied to halide perovskites, which have multiple atoms per unit cell and peaks in the DOS due to mixed character vibrations that cannot be simply categorized by lead or halide atoms alone. Despite this more complicated structure, the simple Einstein oscillator concept using just one average frequency was shown to be a good approximation in MHPs.^31^ Hence, the electron–phonon renormalization can be effectively modeled using a single Einstein oscillator with a positive amplitude, *A*_eff_, to replicate the linear reduction of the gap with decreasing temperature in CsPbBr_3_ NCs. Due to the unknown absolute magnitude of the gap renormalization at room temperature (or any other temperature), we consider instead the temperature derivative of the gap as follows
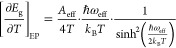
4

**Figure 4:**
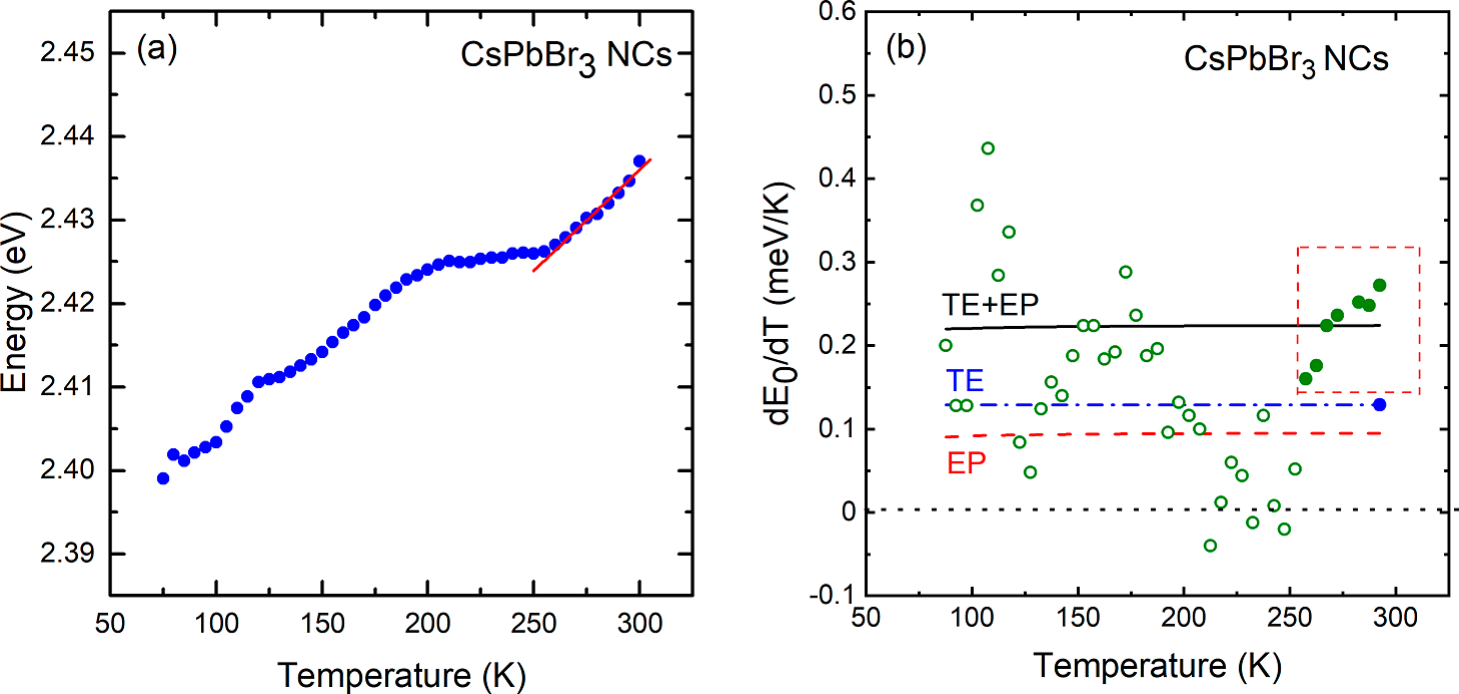
(a) Smoothed data related to the maximum PL peak energies for CsPbBr_3_ NCs plotted as a function of temperature (blue circles) and linearity regime indicated by the red line fitted to the data points around room temperature. (b) First derivative of the PL peak energy *E*_0_ with respect to temperature (green solid and open symbols), numerically obtained from the smoothed data of (a). Only the points shown in the red rectangle (linearity regime) are considered. The different contributions to the band gap renormalization are indicated.

In this way, all the crucial elements to evaluate the weight of electron–phonon coupling in the band gap renormalization are available. Figure 4a presents smoothed data of the PL peak energies as a function of temperature. Data smoothing was performed to prevent undesired amplification of the scatter of the data points during the numerical calculation of the first derivative of *E*_0_ with respect to temperature. The numerically obtained d*E*_0_/d*T* values are displayed in Figure 4b as green circles. Only the green closed circles corresponding to the stability range of the cubic phase are considered during the fitting process (linearity regime). The blue dash-dotted curve in Figure 4b represents the constant contribution of TE term previously calculated as 1.30(40) × 10^–4^ eV/K. Taking this value into account together with the function in eq 4, a fit to the experimental points was performed in the selected temperature range from 250 to 300 K. The adjustable parameters in the EP term determined for CsPbBr_3_ NCs are *A*_eff_ = 6.7(5) meV for the electron–phonon coupling amplitude and *h̵*ω_eff_ = 6(1) meV for the oscillator frequency. The result of the least-squares fit is represented by the black curve. Despite the slight dispersion in the first derivative values, the average slope obtained from the fit (black line) is 2.24(2) × 10^–4^ eV/K, in excellent agreement with the slope of the red curve in Figure 4a.

While the contribution of the EP term near room temperature is clear from the experimental data, its interpretation in terms of eq 4 required to discriminate between multivalued solutions due to the fact that several *A*_eff_ and *h̵*ω_eff_ value pairs can yield the same fit quality. We chose to fix the value of the effective frequency of the Einstein oscillator to 6(1) meV whereas the amplitude *A*_eff_ was kept free in the fitting process. The fixed value of the effective frequency is actually the one previously obtained for MAPbI_3_.^27^ In fact, an inspection of the phonon DOS of bulk MAPbBr_3_, depicted in Figure S4 of the Supporting Information, indicates that the oscillator frequency of 6 meV lies slightly above the strongest low-frequency peak or band in the DOS, corresponding to low-frequency optical modes partially intermixed with acoustical branches.^34^ Hence, the EP term represented by the single Einstein oscillator accounts only for the coupling with the phonons of the inorganic cage. In addition, we note that this value is also close to the effective phonon frequency involved in the exciton broadening as a function of temperature in 11 mixed cation FA_*x*_MA_1–*x*_PbI_3_ solid solutions.^37^ All this supports the idea that the frequency value selected here is quite representative of the EP coupling to the inorganic cage phonons, hence holding for most of the perovskites.

Despite dealing with pure CsPbBr_3_ NCs, the fact that the EP term is fully accounted for with a single Einstein oscillator representing the inorganic cage phonons means that there is no hint to an anomalous EP coupling due to Cs dynamics. This is in frank contrast to the mentioned case of Cs_*x*_MA_1–x_PbI_3_ single crystals (*x* = 0.05 and 0.1), where Cs-substitution leads to an anomalous EP due to the indirect coupling between the charge carriers of the inorganic cage and the fast translational dynamics of the Cs cations.^32^ This anomalous indirect coupling occurs when the dynamic tilting of the PbI_6_ octahedra occurs in synchrony with the motion of the Cs cations between the potential minima within the cage voids. In the case of the CsPbBr_3_ NCs, dynamic octahedral tilting fluctuations centered at zero tilt, as is mandatory for a cubic phase. Consequently, although the dynamics of the Cs cation is unfolded, the motion is restricted to the central region of the cage voids and is characterized by a spherical atomic probability density cloud. In this situation, coherence is simply forbidden by symmetry, so only normal EP coupling remains but no anomalous EP coupling.

We now will show that the behavior observed here, featuring a normal EP coupling term, is general and holds for most of the MPHs. The pressure and temperature dependence of the gap of different MPHs is summarized in Table 1. We list experimental results on the temperature and pressure coefficient of the fundamental gap obtained for lead halide perovskites with formula APbX_3_ in single, poly and nanocrystalline form as well as thin films. It is important to note that the data are circumscribed to near ambient conditions, for which most of the materials crystallize either in a cubic or tetragonal phase. This corresponds here to the linearity regime considered for the analysis of the TE and EP contributions to the gap renormalization. In these phases, static octahedral tilting does not play any role and the gap varies linearly with temperature. Otherwise, strong nonlinearities would affect the temperature and pressure behavior of the perovskite gap. Close inspection of the tabulated data allows for drawing a few interesting conclusions. On one hand, all pressure coefficients are negative, without exceptions, with values bunching between ca −30 and −70 meV/GPa. On the other hand, except for CsPbCl_3_ and (MHy)PbBr_3_, the linear temperature coefficients are positive and in the range of 0.15 and 0.4 meV/K.

**Table 1:** Linear Pressure d*E*_g_/d*P* and Temperature d*E*_g_/d*T* Coefficients of the Fundamental Band Gap, Mainly in the Case of the Tetragonal or Cubic Phase, Stable at Ambient Conditions, Measured for a Series of Lead Halide Perovskite Materials^a^

material		(meV/GPa)	(meV/K)	references
MAPbI_3_	SC	–50(10)	0.26(5)	(31)
	SC	–65(10)*		(67)
	SC	–50(15)*		(49)
	SC	–70(10)*		(51)
	PC	–62(5)		(68)
	PC	–210(50)*		(69)
	TF		0.29(5)	(70)
	TF		0.24(5)	(71)
	TF		0.1(1)	(72)
	NC^>^	–43(6)	0.21(2)	(73)
	NC^<^	–8(7)	0.19(3)	(73)
	NC		0.16(6)	(15)
MAPbBr_3_	SC	–54(5)	0.24(5)	(9)
	SC		0.27(5)	(74)
	SC	–85(15)		(75)
	PC	–52(8)		(51)
	TF		0.14(6)	(71)
	TF		0.15(8)	(72)
	NC		0.33(8)	(76)
	NC		0.36(6)	(18)
	NC		0.44(10)	(77)
MAPbCl_3_	PC	–77(5)		(78)
MA_0.2_FA_0.8_PbI_3_	SC	–54(6)	0.35(10)	(42)
MA_0.3_FA_0.7_PbBr_3_	TF	–41(5)	0.20(10)	(39)
MA_0.3_FA_0.7_Pb_0.5_Sn_0.5_Br_3_	TF	–82(5)	0.30(10)	(39)
MA_0.4_FA_0.6_PbI_3_	SC	–55(5)	0.25(3)	(42)
MA_0.6_FA_0.4_PbI_3_	SC	–58(10)	0.20(10)	(42)
MA_0.8_FA_0.2_PbI_3_	SC	–46(5)	0.27(5)	(42)
MA_0.9_Cs_0.1_PbI_3_	SC	–65(15)	0.11(5)	(32)
MA_0.95_Cs_0.05_PbI_3_	SC	–65(15)	0.13(5)	(32)
MA_0.13_EA_0.87_PbBr_3_	SC	–61(15)	2.81(5)	(79)
FAPbI_3_	SC		0.41(5)	(42)
	TF		0.55(5)	(72)
	TF		0.38(5)	(80)
	NC		0.23(6)	(14)
FAPbBr_3_	SC		0.45(5)	(74)
	SC	–210(20)*		(81)
	TF		0.44(6)	(71)
	TF		0.40(5)	(72)
	NC		0.29(6)	(21)
(MHy)PbBr_3_	SC	–58(15)	–1.97(5)	(79)
CsPbI_3_	NC		0.06(5)	(25)
	NC	–20(10)		(26)
	NC		0.25(5)	(23)
	NC		0.25(5)	(82)
CsPbBr_3_	SC	–72(5)		(83)
	SC	–37(10)		(84)
	SC		0.07(10)	(74)
	SC	–35(5)		(85)
	SC	–34(15)	0.31(5)	(79)
	NC^>^	–55(15)	0.23(5)	this work
	NC		0.05(5)	(25)
	NC^>^	–40(10)		(27)
	NC^<^	–15(10)		(27)
	NC^>^		0.18(5)	(86)
	NC^<^		0.27(5)	(86)
	NC^<^		0.14(6)	(87)
	NC		0.33(8)	(23)
	NC		0.07(10)	(88)
	NC		0.08(10)	(28)
CsPb(I,Br)_3_	NC		0.18(5)	(23)
CsPbCl_3_	TF		–0.15(10)	(89)
	TF		–0.27(10)	(89)
	NC		–0.07(5)	(25)

a The notation SC, PC, TF, and NC stands for single crystal, polycrystal, thin film and nanocrystal, respectively, indicating the structural nature of the samples. For NCs the symbol “<” means average sizes less than 6 nm, “>” means sizes between 6 and 10 nm, and if nothing is indicated then the average NC size is larger than 10 nm. Numbers in parentheses are error bars. The asterisk denotes slope values obtained from only three data points available.

In the following, it will be shown that both trends are not fortuitous but a direct consequence of the bonding/antibonding and atomic orbital character of the electronic states at the top and bottom of the valence and conduction band, shared by all the tabulated materials. Regarding the gap pressure coefficient, it has been shown for the archetypal perovskite MAPbI_3_^43^ that the pressure-induced red shift of the gap in the tetragonal phase, stable at ambient conditions, can be accounted for in terms of the well-established systematics on the pressure dependence of direct band gaps in tetrahedrally bonded semiconductors.^46^ In short, for conventional semiconductors with predominantly sp^3^ hybridization holds that

(a) States with bonding p-orbital character are almost insensitive to pressure. The best example is the top of the valence band at the Γ point of the Brillouin zone (BZ), which is bonding pure p-type.
(b) On the contrary, antibonding s-states exhibit a strong blue shift with pressure. In most of the cases, the conduction band minimum also at Γ comprises states with antibonding pure s-orbital character. For these reasons, in conventional semiconductors, direct gaps at the BZ center increase with pressure at a rate on the order of 100 meV/GPa.^46^
(c) Antibonding p-orbitals are characterized by a much smaller but negative deformation potential as compared with s-states, like conduction band states of the X-valleys at the BZ edge. As a result, the indirect Γ–X gap decreases with pressure at a slower pace of typically ca −15 meV/GPa.^46^

The situation is dramatically different for lead halide perovskites due to the huge spin–orbit splitting of the p-states in heavy atoms like Pb, which leads to a so-called band inversion. Without spin–orbit, the level ordering of s and p atomic orbitals in Pb is similar to that of α-Sn (see Figure 2.26 in ref (61)). Spin–orbit coupling, though, strongly splits the p-states of Pb, pushing the bonding p-states below the energy of the antibonding s-states, such that the atomic orbital character of valence and conduction band states are interchanged, as compared to conventional semiconductors. Relativistic band-structure calculations^62−64^ for a pseudocubic phase of MAPbI_3_ predict a direct fundamental gap at the R-point of the BZ with the top of the valence band predominantly composed by antibonding Pb 6s orbitals hybridized with I 5p orbitals and the bottom of the conduction band formed by the antibonding split-off Pb 6p-orbitals. With increasing pressure one thus expects that the top of the valence band shifts up and the bottom of the conduction band shifts slightly down, leading to a reduction of the fundamental gap, as experimentally observed for all lead halide perovskites (see Table 1). As a corollary of the preceding discussion about the gap pressure coefficient, it results that in perovskites the TE term will always lead to a gradual opening of the gap with increasing temperature. Around ambient conditions, where the pressure coefficient as well as thermal expansion coefficient and bulk modulus are temperature independent, the contribution of thermal expansion to the temperature renormalization of the gap is thus a linear function with a positive slope (constant positive derivative). Since the gap temperature coefficient exhibits a clear systematic regarding sign and magnitude across most lead halide perovskites too, we are led to the conclusion that the contribution of the electron–phonon interaction to the gap renormalization is also determined by the bonding/antibonding and atomic orbital character of the electronic states of the band extrema. Such a relationship, although less transparent than for the pressure coefficient, has been elucidated for tetrahedrally bonded semiconductors in the case of a deformation-potential mediated electron–phonon interaction.^57,65,66^ A similar line of argumentation has been followed to successfully explain the contribution of the EP term to the temperature renormalization of the gap in MAPbI_3_.^31^

## Conclusions

In this study, we investigated the energy gap behavior in CsPbBr_3_ NCs as a function of temperature and pressure. We found that the temperature dependence of the band gap in the temperature range from 250 to 300 K is linear with a positive slope of 2.24(2) × 10–^4^ eV/K. By employing high-pressure experiments, the pressure coefficient was acquired. According to these experiments and using the volumetric thermal expansion coefficient and bulk modulus of CsPbBr_3_ from the literature, contributions of TE and EP to the gap renormalization were disentangled. The obtained value of TE term is 1.3(4) × 10^–4^ eV/K and EP contribution is 1.0(3) × 10^–4^ eV/K, which demonstrates that both terms have significant and almost equal weight in the gap renormalization. A single Einstein oscillator model was used to assess the effects of electron–phonon coupling on the temperature renormalization of the gap. This oscillator with positive amplitude, which accounts solely for the coupling with phonons of the inorganic cage, is all what is needed (apart from thermal expansion effects) for an accurate description of the linear gap temperature dependence around room temperature. Interestingly, an exhaustive survey among lead halide perovskites comparing their gap temperature and pressure coefficients shows that this is the most common situation, whereas that of an indirect electron–phonon coupling involving degrees of freedom of the A-site cations is clearly exceptional, i.e. just an anomaly. Finally, we note that although our analysis is restricted to a limited temperature range imposed by the linearity extension, the fact that this occurs around room temperature is particularly relevant for both photovoltaic and lighting applications. Solar cells and LEDs operate in this temperature regime and a clear understanding of the cause and magnitude of the change in the gap with temperature is crucial for optimization purposes. In this way, we were able to shed light on a fundamental issue, that of the temperature renormalization of the gap of lead halide perovskites, a key information in optoelectronics.

## Data Availability

All data generated or analyzed during this study are either included in this published article and its Supporting Information files or are available from the corresponding author on reasonable request.

## Supporting Information Available

Contains results of the TEM and Raman characterization of the CsPbBr_3_ NCs that indicate the cubic phase is the stable one at ambient conditions. Besides, the PL spectra recorded at different pressures are also presented. The maximum of the PL peaks gave the energy gap for each pressure and accordingly the pressure coefficient was obtained. Finally, a detailed discussion of the meaning of the parameters entering the Einstein-oscillator model is provided (PDF)
