# Supplementary material for: Absence of Anomalous Electron–Phonon Coupling in the Near-Ambient Gap Temperature Renormalization of CsPbBr3 Nanocrystals
Source: J Phys Chem C Nanomater Interfaces. 2024 Dec 19;129(1):453–63. doi: 10.1021/acs.jpcc.4c06265 (PMC11921149; doi:10.1021/acs.jpcc.4c06265)
Supplement: Supplementary file 1 — jp4c06265_si_001.pdf [file jp4c06265_si_001.pdf]

Supporting information

Absence of Anomalous Electron-Phonon Coupling in the Near-Ambient Gap Temperature Renormalization of $CsPbBr_3$ Nanocrystals

Shima Fasahat[1], Benedikt Schäfer[1], Kai Xu[1], Nadesh Fiuza-Maneiro[2], Sergio Gómez-Graña[2], M. Isabel Alonso[1], Lakshminarayana Polavarapu[2], Alejandro R. Goñi[1,3*]

[1]Institut de Ciencia de Materials de Barcelona, ICMAB-CSIC, Campus UAB, 08193 Bellaterra, Spain
[2]CINBIO, Universidade de Vigo, Materials Chemistry and Physics Group, Dept. of Physical Chemistry, Campus Universitario Lagoas Marcosende, 36310 Vigo, Spain
[3]ICREA, Passeig Lluís Companys 23, 08010 Barcelona, Spain
*Corresponding author: goni@icmab.es

Section S1: TEM characterization

The structural characterization of the $CsPbBr_3$ nanocrystals (NCs) was performed using high-resolution TEM, which indicates that the sample is fairly crystalline and the NCs present ordered cubic shapes. The TEM images with lower magnification allowed us to measure the side lengths of the nanocubes precisely, sampling over hundreds of NCs. A representative histogram is shown in Fig. S1, displaying the NCs size distribution that was obtained by considering 126 $CsPbBr_3$ NCs. The NC ensemble exhibits a relatively narrow size distribution with an average edge length of 8 nm.

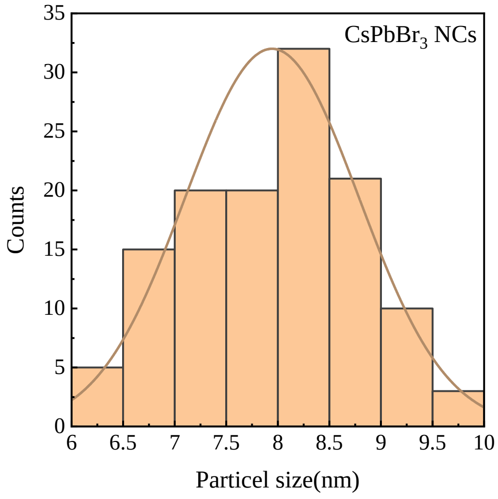

Figure S1: Histogram of the size distribution of $CsPbBr_3$ NCs, constructed from TEM micrographs containing more than hundred NCs.

Section S2: Raman scattering measurements

A representative Raman spectrum of $CsPbBr_3$ NCs recorded at ambient pressure and temperature conditions is shown in Fig. S2. Typical of the cubic phase, for which the Cs dynamics is known to be fully unfolded, is the steep increase in Raman intensity for very small Raman shifts, due to the presence of a “zero-shift” peak. This peak arises from incoherent scattering due to fluctuations in the Raman susceptibility as a consequence of the unleashed Cs dynamics and its effect on the inorganic cage phonons [1]. Besides, three broad bands can be barely resolved on top of the steep slope, in the spectral range of the inorganic cage phonons below 200 cm^{-1}. The strong broadening of the Raman peaks is additional evidence of the presence of dynamic disorder, a characteristic feature of the cubic phase [2,3].

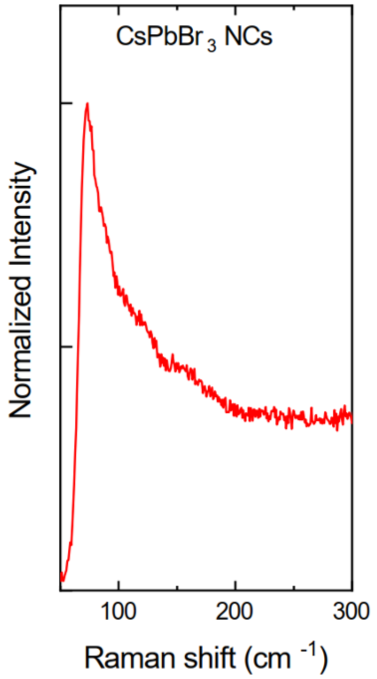

Figure S2: Room-temperature Raman spectrum of $CsPbBr_3$ NCs recorded using the infrared (785 nm) laser line for excitation.

Section S3: PL measurements under pressure

Representative photoluminescence (PL) spectra of $CsPbBr_3$ NCs measured at different pressures at room temperature are shown in Fig. S3. Initially, the main PL peak shifts to the red with increasing pressure. The line shape changes observed in the PL spectra starting at 0.91 GPa are a consequence of the occurrence of a pressure-induced first-order phase transition [4]. Within a certain range around the phase transition pressure, the coexistence of the two crystal phases is observed (double PL peak). To calculate the TE term of the gap temperature dependence, the

pressure coefficient of the gap is obtained only from the PL peak positions of the spectra from the phase stable at ambient conditions.

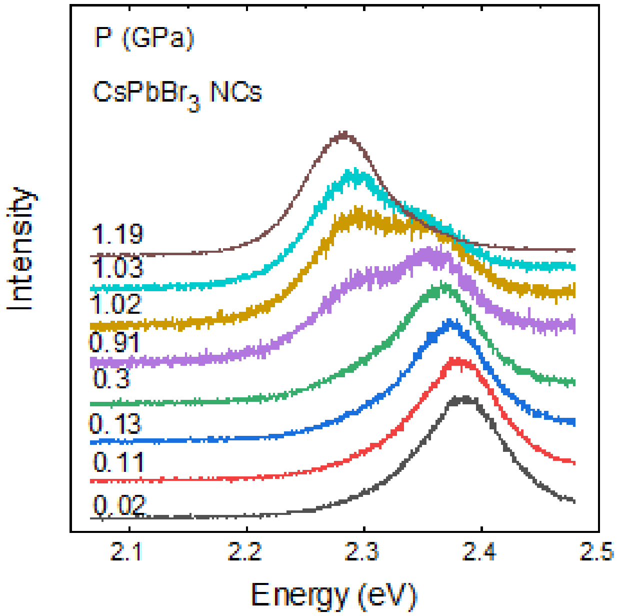

Figure S3: PL spectra of CsPbBr3 NCs under different pressures.

The two terms of Eq. (1) of the manuscript, which defines the derivative of the gap over temperature, can be rewritten in the following way:

$$\frac{dE_g}{dT} = \left.\frac{\partial E_g}{\partial V}\right|_T \cdot \left.\frac{\partial V}{\partial T}\right|_P + \left.\frac{\partial E_g}{\partial T}\right|_V . \qquad (1)$$

The first term accounts for thermal expansion effects (TE) by considering the change of the gap due to a change in volume but at constant temperature, multiplied by the change in volume with temperature at constant pressure. The latter is related to the volumetric thermal expansion coefficient α_V. The second term in Eq. (1) corresponds to the gap renormalization directly caused by electron-phonon interaction (EP) and is given by the variation of the gap over temperature at constant volume. Taking into account that the bulk modulus B_0 is the inverse of the compressibility, the thermal expansion term can be further rewritten in terms of the pressure differential as:

$$\left.\frac{\partial E_g}{\partial V}\right|_T \cdot \left.\frac{\partial V}{\partial T}\right|_P = \left.\frac{\partial E_g}{\partial P}\right|_T \cdot \left.\frac{\partial P}{\partial V}\right|_T \cdot \left.\frac{\partial V}{\partial T}\right|_P \cdot \frac{V}{V} \tag{2}$$

$$= \underbrace{\frac{1}{V}\left.\frac{\partial V}{\partial T}\right|_P}_{\alpha_V} \cdot \underbrace{V \left.\frac{\partial P}{\partial V}\right|_T}_{-B_0} \cdot \left.\frac{\partial E_g}{\partial P}\right|_T . \tag{3}$$

The thermal expansion term finally is given as:

$$\left[\frac{\partial E_g}{\partial T}\right]_{TE} = -\alpha_V \cdot B_0 \cdot \underbrace{\left.\frac{\partial E_g}{\partial P}\right|_T}_{initial\ slope} , \tag{4}$$

where initial slope means that the derivative must be computed near ambient pressure (P→0).

Section S4: Single Einstein-oscillator model

Here the EP coupling term is described in terms of a single Einstein-oscillator model, which contains two parameters: The oscillator amplitude A_{eff} and its characteristic frequency ω_{eff}. In principle, the latter should correspond to a given peak in the phonon density of states (DOS). For that purpose we reproduce the calculations of the phonon dispersion curves and the corresponding DOS from Ref. [2]. The vibrational frequencies for the cubic phase of $MAPbBr_3$ were calculated within the harmonic phonon approximation using second order force constants obtained from density functional theory (DFT). Figure S4 (left panel) shows the phonon dispersions of the 18 inorganic cage modes within the harmonic approximation using a pseudo cubic lattice for $MAPbBr_3$ at room temperature. Negative-frequency *soft* modes are located around the Brillouin-zone boundary at the M and R points. The phonon dispersions are plotted considering band crossings. The color refers to the nature of each phonon eigenmode. The three orthogonal acoustic modes are plotted in blue shades. The remaining modes are optical, plotted in groups of three with a similar shade for each orthogonal mode (these modes would be degenerate if the MA ions were replaced by a spherical atom). Figure S4 (right panel) displays the corresponding phonon DOS decomposed by sets of three orthogonal phonon eigenmodes, integrated over the full Brillouin zone, but not considering band crossings.

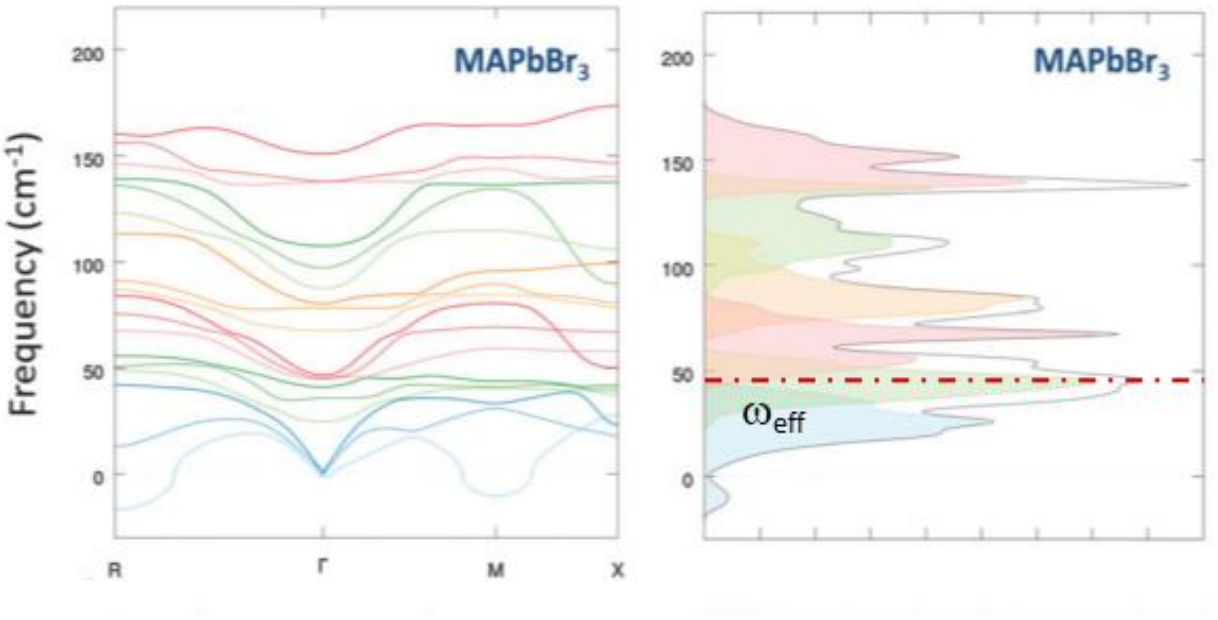

Figure S4: (Left panel) Phonon dispersion curves and (Right panel) phonon density of states (DOS) of bulk $MAPbBr_3$ calculated within the harmonic phonon approximation using second order force constants obtained from density functional theory (adapted from Fig. 3 of Ref. [2]).

The dot-dashed horizontal line in the right panel of Fig. S4 represents the effective frequency of the Einstein oscillator used to describe the EP coupling term in the case of the $CsPbBr_3$ NCs. An inspection of the phonon DOS indicates that the oscillator frequency of 6 meV (i.e. ca. 48 cm^{-1}) lies slightly above the first well-defined peak or band in the DOS, corresponding to low-frequency optical modes partially intermixed with acoustical branches. Since these are all phonons of the inorganic cage, we can safely assume that a similar oscillator will account for the EP term in the $CsPbBr_3$ NCs as well. This is so because both $MAPbBr_3$ and $CsPbBr_3$ share the same inorganic cage, being the influence of the A-site cation negligible to first order of approximation. Furthermore, we note that the contributions of the higher-frequency peaks in the phonon DOS are exponentially damped by the Bose-Einstein phonon occupation number. This explains why a single Einstein oscillator is enough to account for the coupling between the electrons and inorganic cage phonons.

References

[1] Yaffe, O.; Guo, Y.; Tan, L. Z.; Egger, D. A.; Hull, T.; Stoumpos, C. C.; Zheng, F.; Heinz, T. F.; Kronik, L.; Kanatzidis, M. G.; et al. Local Polar Fluctuations in Lead Halide Perovskite Crystals. *Phys. Rev. Lett.* **2017**, 118, 136001.

[2] Leguy, A. M. A.; Goñi, A. R.; Frost, J. M.; Skelton, J.; Brivio, F.; Rodríguez-Martínez, X.; Weber, O. J.; Pallipurath, A.; Alonso, M. I.; Campoy-Quiles, M.; Weller, M. T.; Nelson, J.; Walsh, A.; Barnes, P. R. F. Dynamic Disorder, Phonon Lifetimes, and the Assignment of Modes to the Vibrational Spectra of Methylammonium Lead Halide Perovskites. *Phys. Chem. Chem. Phys.* **2016**, 18, 27051-27066.

[3] Goñi, A. R. Raman Linewidths as a Probe of Lattice Anharmonicity and Dynamic Disorder in Metal Halide Perovskites. *Asian J. Phys.* **2024**, 33, 29-38.

[4] Beimborn, J.C.; Walther, L.R.; Wilson, K.D.; Weber, J.M. Size-dependent pressure-response of the photoluminescence of $CsPbBr_3$ nanocrystals. *J. Phys. Chem. Lett.* **2020,** 11, 1975-1980.
